# The Differential Role of Cytokines on Stress Responses in a Menopause Rat Model

**DOI:** 10.3389/fpsyt.2020.577561

**Published:** 2020-11-19

**Authors:** Hyun Jung Park, Hyun Soo Shim, Insop Shim

**Affiliations:** ^1^Department of Physiology, College of Medicine, Kyung Hee University, Seoul, South Korea; ^2^Department of Food Science and Biotechnology, Kyonggi University, Suwon, South Korea

**Keywords:** cytokines, depression, motor trigeminal nucleus (MTN), para ventricular nucleus (PVN), ovariectomy

## Abstract

Menopause is a risk factor of anxiety and depression. Also, psychoneurological symptoms are shown in almost all women in the perimenopausal period. The present study investigated if repeated stress modulates behavioral changes or the balance of pro- and anti-inflammatory cytokines in ovariectomized (OVX) rats. Albino SD female rats were randomly divided into four groups: the naïve normal group (NOR), a surgically ovariectomized group (OVX), the only stressed group (ST), and the OVX and stressed groups (OVX + ST). We performed a battery of tests such as the forced swimming test (FST), the sucrose intake, and social exploration. In the same animals, corticosterone (CORT) was assessed in the serum, and also, two representative cytokines (IL-1β and IL-4) were examined in different brain regions after all the behavior sessions for all the experimental groups. The OVX + ST group showed more immobility time in FST than the OVX group or the ST group. Also, the OVX + ST group tended to have a decreased active social exploration and sucrose solution intake compared to the OVX group or ST group. The serum concentration of CORT of the OVX + ST group was higher than the OVX group or ST group and also the level of CORT in OVX + ST was markedly increased compared to the NOR group. In the brain, the number of IL-1β immunoreactive neurons of the OVX + ST group was increased compared to the NOR group. The OVX + ST group tended to have an increase in IL-1β-positive neurons compared to the OVX or ST group. However, the number of IL-4 immunoreactive neurons of the OVX + ST group was markedly decreased compared with the NOR group. Also, the IL-4-positive neurons in the OVX + ST group was significantly decreased when compared to the ST group. These results indicate that ovariectomy and stress combine to increase the depressive-like behaviors and neuroinflammatory responses. Together, these data show neuroinflammation as a potential contributor to depressive-like symptoms during menopausal transition.

## Introduction

Ovarian hormones play a pivotal role in regulating affective disorder (1). Depletion of ovarian hormone as menopausal implicated increased mood disorders and deficient learning and memory (2). Also, several studies proved that estrogen and testosterone regulate HPA axis responses (3, 4). The postmenopausal stage in women exacerbates mood disorder or insomnia (5). Some studies implicated that mood disorders are related to change of neuroimmune systems (6–9). For example, chronic restraint stress or foot shock stress elicits depressive behaviors (learned helplessness, anhedonia, anxiety, etc.) and also chronic stress alter neuroimmune systems (10, 11). Other studies reported that psychological stress is associated with pro-inflammatory cytokine release or depressive behavior (12, 13). In a recent study, infectious challenge with IL-1β to IL-4 KO mice resulted in dysregulation of pro-inflammatory cytokines (14). IL-4 KO is capable of causing susceptibility to immune responses.

A recent study proved that the postmenopausal stage altered the neuroendocrine–immune system (15). Menopausal women experience difficulty in coping with stressful situations. Stress levels are perceived to vary with menopause (16). This is related to pro-inflammatory cytokine levels in the serum (16). Also, the ovarian hormone exerts potent immunomodulatory effects in neuroinflammation animal models (17–19). Although estrogen is associated with menopause-related neuronal inflammation changes, the mechanisms behind its effects are unclear.

In this study, we aimed to investigate whether repeated stress in the OVX rat model could change depressive-like behaviors and changes of pro- and anti-inflammatory cytokines in the brain regions. Depressive behavior was tested via a forced swimming test (FST), social exploration, and sucrose intake; moreover, we further assessed the changes of IL-1β- and IL-4-reactive neurons in the PVN and MTN using immunohistochemistry.

## Materials and Methods

### Animals and Immobilization Stress

Albino SD female rats (over 3 months old, weighing 240–300 g) (Orient, Inc. Korea) underwent ovariectomy. All rats were housed in a plastic cage with stainless steel lips under controlled conditions (temperature: 22–24°C, 12-h light/dark cycle, and humidity 65 ± 5%). The rats had *ad libitum* access to food and water. All the experiments were approved by the Kyung Hee University Institutional Animal Care and Use Committee [approval no. KHUAP(SE)-13-041].

Albino SD female rats were randomly divided into four groups: the naïve normal group (NOR, *n* = 6), a surgically ovariectomized group (OVX, *n* = 6), the only stressed group (ST, *n* = 6), and the OVX and stressed group (OVX + ST, *n* = 6). Bilateral ovariectomy was performed under pentobarbital sodium (50 mg/kg, i.p.). The animals were monitored for 1 week post-surgery. Postoperative recuperation was monitored for 1 week.

**Figure 1:**
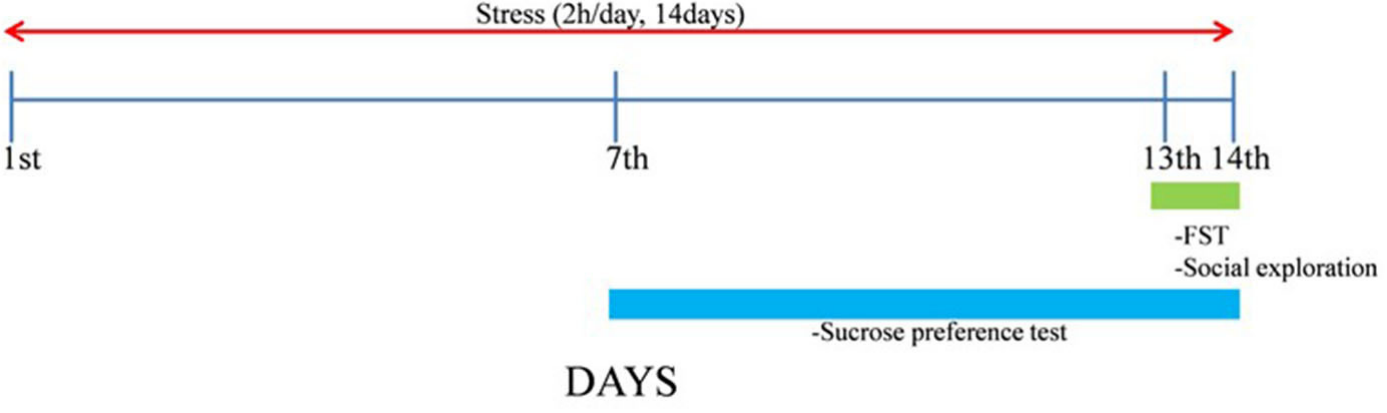
Experimental schedule. The naïve normal group (NOR), a surgically ovariectomized group (OVX), the only stressed group (ST), and the OVX and stressed group (OVX + ST). Each group contained six rats (*n* = 6).

After 1 week, ST groups were exposed to repeated immobilization stress using the disposable plastic cone (a disposable rodent restraint cone, Yusung, Korea) for 2 h (10:00–12:00 a.m.) for 14 days. The experimental schedule is shown in Figure 1.

### Forced Swimming Test

Transparent Plexiglas cylinders (height: 50 cm × diameter: 20 cm) were used for FST. The room temperature water was filled to a 30-cm depth. Before test, all rats were taken pre-test for 15 min. After 24 h, rats were tested for 5 min. We used a video camera and analyzed. Total duration of immobility, climbing, and swimming were examined (20).

### Social Exploration

On the first day, rats were introduced into their transparent home cage for 5 min. After 24 h, rats were recorded as doing “social exploration” for 5 min. We counted the behaviors (active behaviors: grooming of test rat, sniffing, and liking; inactive behaviors: boxing and kicking).

### Sucrose Preference Test

All rats assessed adaptive training for 1 week. Training consisted of seven 12-h tests, and rats could freely choose between a bottle of water and a bottle containing a mild 1% [weight/volume (w/v)] sucrose solution. Two bottles were rotated daily. Each rat freely selected a pre-weighed tap water bottle or a 1% sucrose bottle. On the test day (13th day), the total consumption of each bottle was recorded after 12 h.

### Corticosterone Measurement

The blood samples were collected and then centrifuged. The final supernatant was used for corticosterone analysis. Concentration of corticosterone was determined using ELISA kit (DuoSet ELISA development system, R&D Systems, Inc., Minneapolis, MN., USA) according to the manufacturer's protocols.

### Immunohistochemistry

After all behavioral tests, rats were euthanized with an overdose of sodium pentobarbital (80 mg/kg, IP) and then they were transcardially perfused with phosphate buffered saline (PBS) into a protrusion of the left ventricle, with a steady flow of around 20 ml/min of saline solution. When all blood has been cleared from the body, we used a cold 4% paraformaldehyde solution at a flow rate of 7 ml/min (800 ml). The brains were removed and placed in a 4% paraformaldehyde solution for 24 h at 4°C. The brain was placed in O.C.T. compound at −20°C and cut into 30-μm serial coronal sections. Sections were rinsed with PBS containing Triton X-100. The primary antibody concentrations were at 1:100 and 1:1,000 for IL-1β (Santa Cruz Biotechnology, Delaware Avenue Santa Cruz, CA, USA) and IL-4 (Santa Cruz Biotechnology, Delaware Avenue Santa Cruz, CA, USA), respectively. The primary antibody was diluted with blocking serum in ABC kits (Vector Laboratories, Burlingame, CA) for 72 h at 4°C. Then, tissues were washed three times in PBS. For the secondary antibody use as the appropriate primary antibody (1:200) in PBST. Tissues were washed three times in PBS and stained using diaminobenzidine (DAB) chromogen with nickel intensification and coverslipped slides with mounting media. Light microscopy was used for acquiring photomicrographs. The stained cell was counted in paraventricular nucleus (PVN) and motor trigeminal nucleus (MTN) according to a brain atlas (21).

### Statistical Analysis

SPSS 15.0 software (SPSS Inc., Chicago, IL) was used for statistical analysis. Differences among groups in the behavioral test and immunohistochemistry were analyzed using one-way ANOVA and LSD *post hoc* test. A *P* < 0.05 was considered statistically significant. GraphPad Prism 6.0 software was used for graph generation.

## Results

### Behavioral Tests

**Figure 2:**
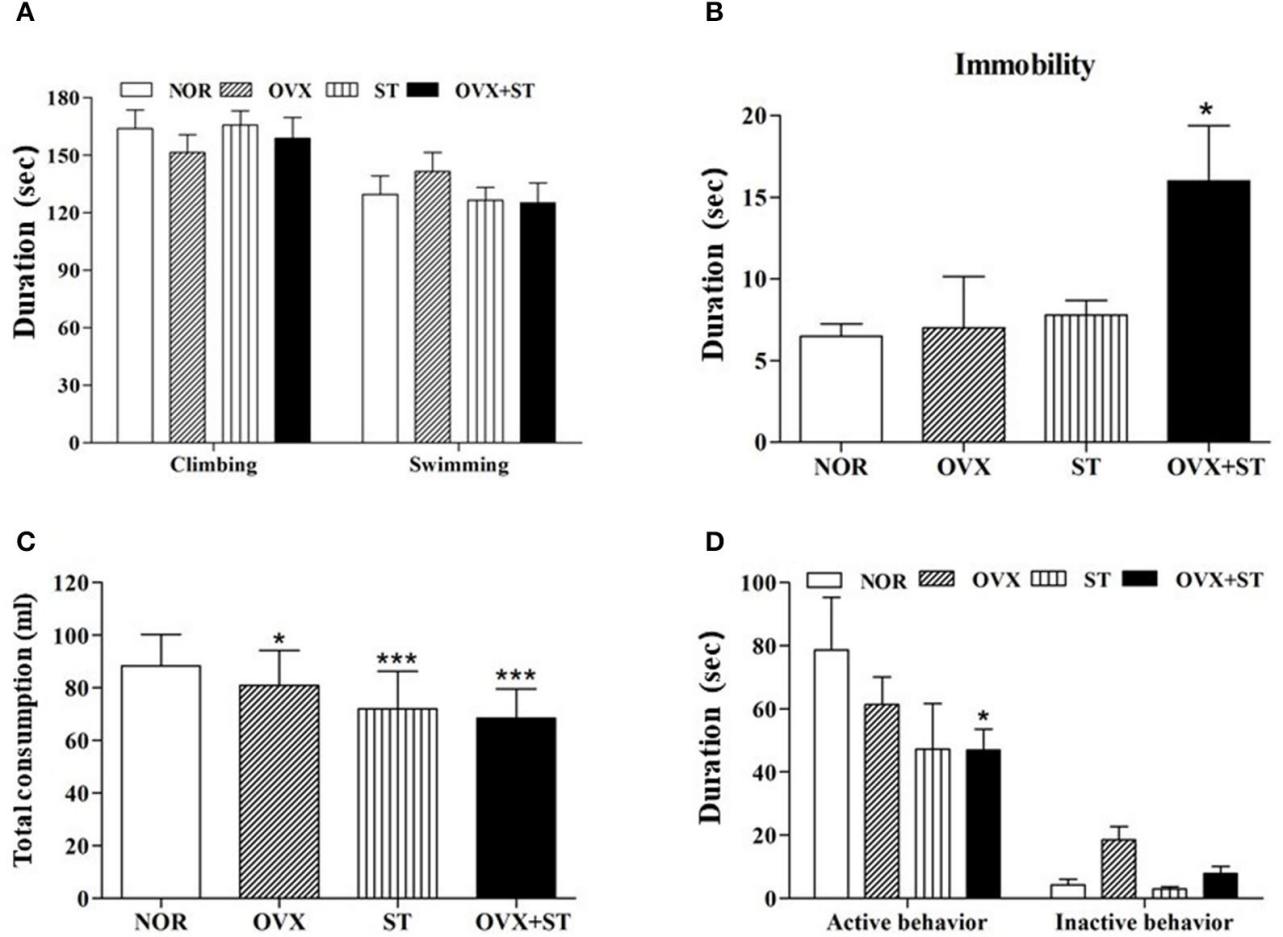
The effects of repeated immobilization stress on the forced swimming test in the rats. The data represent the means ± SEM of the duration of climbing and swimming **(A)** and immobility **(B)** during the 5-min test session. Also, the effects of repeated immobilization stress on the sucrose intake **(C)** and social exploration **(D)**. The data represent the duration of active behavior during the 5-min test session. The naïve normal group (NOR), a surgically ovariectomized group (OVX), the only stressed group (ST), and the OVX and stressed group (OVX + ST). Each group contained six rats (*n* = 6). The results of behaviors were analyzed by performing separate one-way ANOVA among the groups. *p < 0.05, ***p < 0.001 compared to the normal group.

As shown in Figures 2A,B, the immobility time of the OVX groups in the FST significantly increased compared to the NOR group [*F*_(3, 20)_ = 4.7, *P* < 0.05]. When tested in the FST, the OVX and OVX + ST groups tended to show decreased active behavior (swimming and climbing) compared to the NOR group. Figure 2C shows that the sucrose preference test was significantly different when compared among the groups [F(3, 20) = 22.9, *P* < 0.001]. Also, the OVX + ST group showed more immobility time compared to the OVX group or the ST group. Furthermore, the OVX + ST group showed a decrease in consumption of sucrose solution by 30% compared with the NOR group (*P* < 0.001). Also, the OVX + ST group tended to decrease sucrose solution intake compared to the OVX group or the ST group. Figure 2D shows that the social exploration tests were markedly different among the groups [*F*_(3, 20)_ = 8.3, *P* < 0.01]. The active behavior of the juvenile was decreased in the OVX + ST group compared with the NOR group (*P* < 0.05). Also, the OVX + ST group tended to have a decreased active social exploration when compared to the OVX group or the ST group.

### Corticosterone

**Figure 3:**
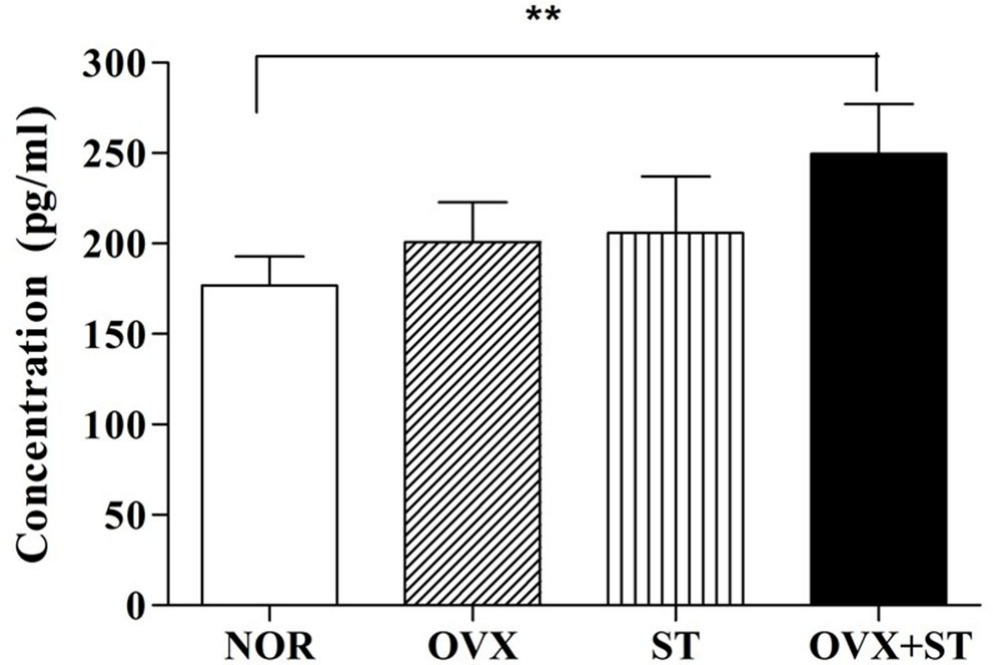
The effects of the corticosterone level in the serum. The naïve normal group (NOR), a surgically ovariectomized group (OVX), the only stressed group (ST), and the OVX and stressed group (OVX + ST). Each group contained six rats (*n* = 6). The results of ELISA were analyzed by performing separate one-way ANOVA among the groups. Each value represents the mean ± S.E.M. **p < 0.01 compared to the normal group.

As shown in Figure 3, the serum concentration of CORT was significantly different among the groups [*F*_(3, 20)_ = 5.8, *P* < 0.01]. Statistical results indicated the markedly increased serum concentration of CORT in the OVX + ST group compared with the NOR group (*P* < 0.01), and the level of CORT in OVX +ST was markedly increased compared to the NOR group.

### IL-1β Immunohistochemistry

**Figure 4:**
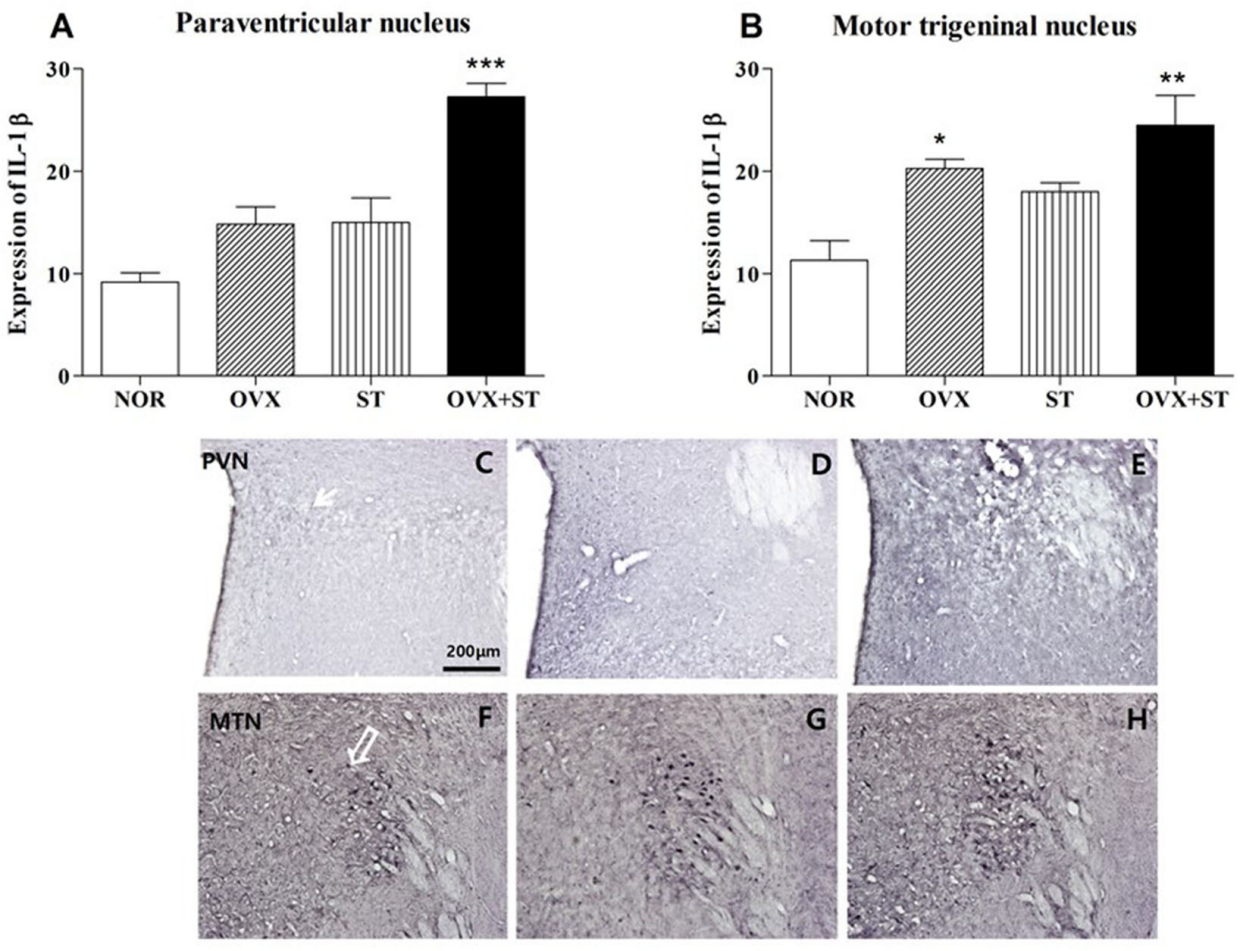
The number of IL-1β immunostained nuclei in the paraventricular nucleus **(A)** and motor trigeminal nucleus regions **(B)**. The naïve normal group (NOR), a surgically ovariectomized group (OVX), the only stressed group (ST), and the OVX and stressed group (OVX + ST). Each group contained six rats (*n* = 6). The results of IL-1β-reactivity were analyzed by performing separate one-way ANOVA on the number of the IL-1β immunostained neurons among the groups. Each value represents the mean ± S.E.M. *p < 0.05, **p < 0.01, ***p < 0.001 compared to the normal group. Photographs showing the distribution of IL-1β immunoreactive cells in the brain of NOR **(C,F)**, OVX **(D,G)**, and OVX + ST **(E,H)**. Sections were cut coronally at 30 μm and the scale bar represents 200 μm. PVN, paraventricular nucleus; MTN, motor trigeminal nucleus.

As shown in Figures 4A–H, the number of IL-1β-positive neurons in the PVN [*F*_(3, 14)_ = 23.7, *P* < 0.001] and MTN (*F*_(3, 14)_ = 9.0, *P* < 0.01) was significantly different among the groups. The expression of the IL-1β-positive neurons in the PVN of the OVX + ST group increased threefold compared with the NOR group (*P* < 0.001). Also, the expression of the IL-1β positive neurons in the MTN was significantly increased in the OVX + ST group compared with the NOR group (*P* < 0.01). The OVX + ST group tended to have an increase in IL-1β positive neurons compared to the OVX or ST group in the MTN and PVN.

### IL-4 Immunohistochemistry

**Figure 5:**
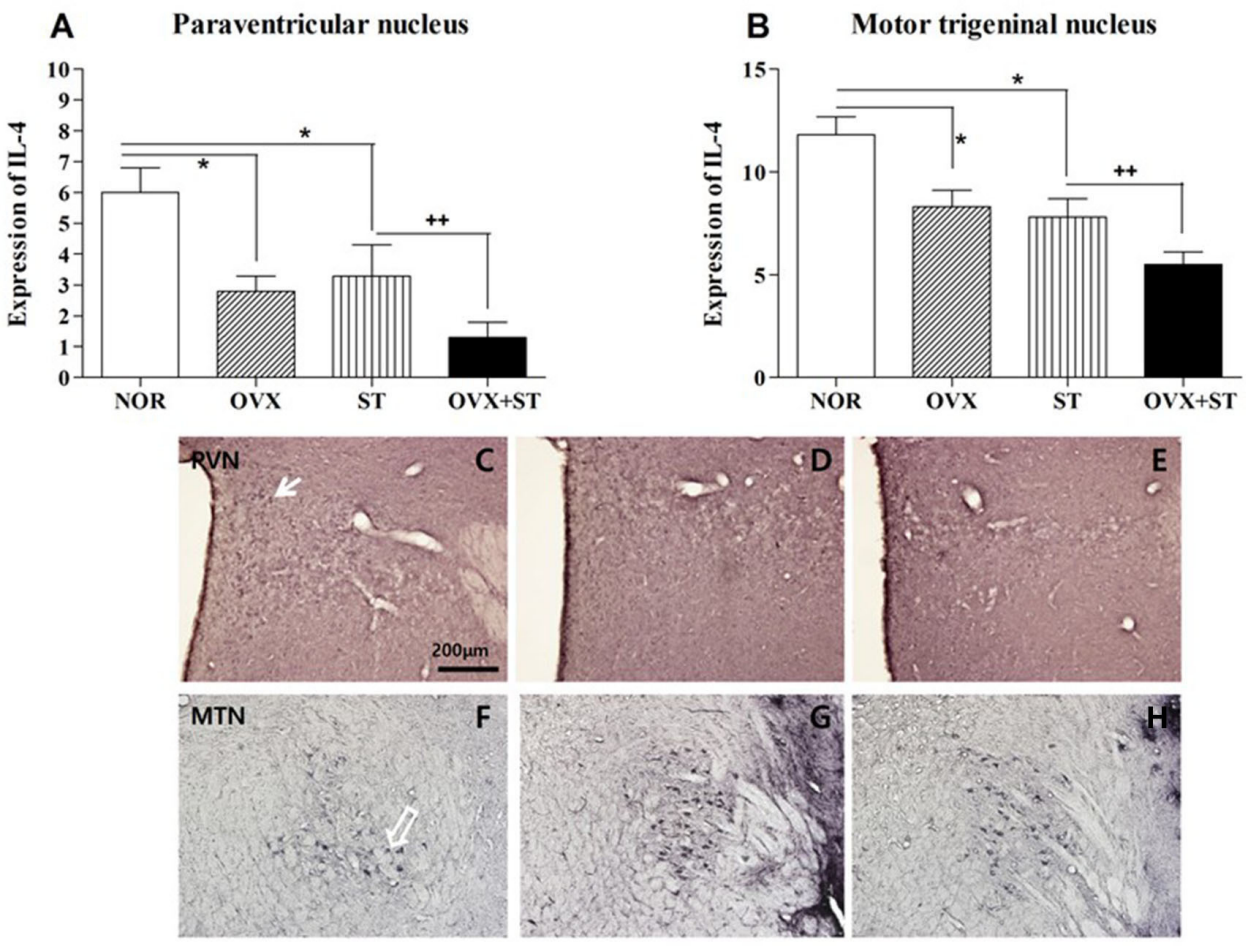
The number of IL-1β immunostained nuclei in the paraventricular nucleus **(A)** and motor trigeminal nucleus regions **(B)**. The naïve normal group (NOR), a surgically ovariectomized group (OVX), the only stressed group (ST), and the OVX and stressed group (OVX + ST). Each group contained six rats (*n* = 6). The results of IL-4 reactivity were analyzed by performing separate one-way ANOVA on the number of the IL-4 immunostained neurons among the groups. Each value represents the mean ± S.E.M. *p < 0.05 compared to the normal group. ^++^*p* < 0.01 compared to the ST group. Photographs showing the distribution of IL-4 immunoreactive cells in the brain of normal group **(C,F)**, OVX **(D,G)**, and OVX + ST **(E,H)**. Sections were cut coronally at 30 μm and the scale bar represents 200 μm. PVN, paraventricular nucleus; MTN, motor trigeminal nucleus.

As shown in Figures 5A–H, the number of IL-1β-positive neurons in the PVN [*F*_(3, 13)_ = 7.2, *p* < 0.05] and MTN [*F*_(3, 15)_ = 11.0, *p* < 0.001] was significantly different among the groups. The number of IL-4 neurons in the PVN area of the OVX + ST group was decreased compared to that in the normal group. Also, the expression of the IL-4-positive neurons in the PVN was significantly decreased in the OVX + ST group compared with the NOR group (*P* < 0.05). In the MTN, the expression of the IL-4-positive neurons was markedly decreased in the OVX +ST group compared with the NOR group (*P* < 0.05). Also, the IL-4-positive neurons in the OVX + ST group was significantly decreased when compared to the ST group.

### The Relationships Between IL-1β and IL-4 Expression

The Spearman's correlation test revealed significant relationships between IL-1β and IL-4. In the experiment groups, biochemical and neuro-immunological characteristics were changed. In particular, the mean levels of IL-1β increased significantly after OVX and repeated stress, whereas IL-4 decreased significantly. In the PVN (*r* = 0.7, *P* < 0.05) and MTN (*r* = 0.7, *P* < 0.01), the change in IL-1β and IL-4 correlates significantly after OVX and repeated stress.

## Discussion

The present results proved that repeated immobilization stress in the OVX rats poses an immune challenge that is capable of inducing depressive like behaviors, promoting exaggerated corticosterone responses and changing the cytokine expression in the brain. The present results showed that the interaction between the anti- and pro-inflammatory mediators ultimately determines both the severity of the manifestation of depressive-like behavior and neuro-immune dysfunction.

Ovarian hormones have played a role in the regulation of cognitive and mood functions (22). They have been proposed as a contributor to depressive behavior and the synthesis, release, and reuptake of many receptors for neurotransmitters (23). This study also showed an increase of depression-like behaviors including reduction of preferences for sucrose solution, inhibition of locomotor activities, and induction of learned helplessness behavior. Also, the effects of female sexual hormones on both neuroinflammatory responses and depressive behaviors have also been well described by Azizi-Malekabadi et al. (22). 17β-estradiol (E2) replacement exhibits anti-inflammatory properties in the central nervous system (18). Overproduction of pro-inflammatory cytokine could be related to the inflammatory response system, which is upregulated during depression, and this is related to the shift of the pro-/anti-inflammatory cytokine balance. A previous study proved that psychological stress is associated with pro-inflammatory cytokines in the brain such as IL-6, interleukin-1β (IL-1β), and tumor necrosis factor α (TNF-α) (24). You et al. proved that chronic mild stress induced inflammatory responses in the spleen and brain region, as well as increased depressive-like behaviors in rats (25). The present study also observed that the expression of IL-1β immunoreactive cells was increased in the PVN and MTN after OVX and repeated immobilization stress. These results indicated that life events and depressive symptoms are related to the rise of central pro-inflammatory cytokines such as IL-1β in major mood disorder patients (26–29) and stress-treated animals (30).

On the other hand, Myint et al. and Pavon et al. reported that depressed patients showed lower IL-4 levels in the serum (28, 31). Also, recent findings in laboratory studies have highlighted the role of interleukin-4, which is released from glia (32), in regulating the neuroinflammatory changes that occur in brain regions. We also observed that the IL-4 immunoreactivity was downregulated in the brain after repeated immobilization stress. The data in this current study indicate that there is an imbalance between anti-inflammatory and pro-inflammatory cytokines (IL-4 and IL-1β) in depressed female rats. The shift between anti-inflammatory and pro-inflammatory cytokines in different psychiatric disorders has been previously reported, and the dominance of macrophage cytokines in major depression has also been described (28). The present study showed that the increased IL-1β/IL-4 ratio may be associated with the activation of macrophage-induced inflammatory response. The present study also found that stimulated production of IL-1β and IL-4 displayed an opposite inflammatory pattern. These changes could be part of normal immune modulation. According to present results, we found an imbalance between anti-inflammatory and pro-inflammatory cytokines in depressed female rats that is associated with life events.

## Data Availability Statement

The datasets generated for this study are available on request to the corresponding author.

## Ethics Statement

All the experiments were approved by the Kyung Hee University Institutional Animal Care (Approval No. KHUAP(SE)-13-041) and in accordance with the US National Institutes of Health Guide for the Care and Use of Laboratory Animals (NIH Publication number 80-23, revised 1996).

## Author Contributions

IS, HS, and HP designed the study. HP and HS acquired and analyzed the data. HP, HS, and IS wrote the article, which all other authors reviewed. All authors contributed to the article and approved the submitted version.

## Conflict of Interest

The authors declare that the research was conducted in the absence of any commercial or financial relationships that could be construed as a potential conflict of interest.
